# *Nowcast-It:* A Practical Toolbox for Real-Time Adjustment of Reporting Delays in Epidemic Surveillance

**DOI:** 10.3390/v17121598

**Published:** 2025-12-10

**Authors:** Amna Tariq, Ping Yan, Amanda Bleichrodt, Gerardo Chowell

**Affiliations:** 1 Department of Pediatrics Infectious Diseases, Stanford School of Medicine, Palo Alto, CA 94305, USA; 2 Department of Statistics and Actuarial Science, University of Waterloo, Waterloo, ON N2L 3G1, Canada; 3 Department of Public Health Sciences, Clemson University, Clemson, SC 29634, USA; 4 Department of Population Health Sciences, Georgia State University, Atlanta, GA 30303, USA; 5 Department of Applied Mathematics, Kyung Hee University, Yongin 17104, Republic of Korea

**Keywords:** nowcasting, reporting delays, survival analysis, non-stationary reporting delays, toolbox, shiny app

## Abstract

One difficulty that arises in tracking and forecasting real-time epidemics is reporting delays, which are defined as the inherent delay between the time of symptom onset and the time a case is reported. Reporting delays can be caused by delays in case detection, symptom onset after infection, seeking medical care, or diagnostics, and they distort the accurate forecasting of diseases during epidemics and pandemics. To address this, we introduce a practical nowcasting approach grounded in survival analysis and actuarial science, explicitly allowing for non-stationarity in reporting delay patterns to better capture real-world variability. Despite its relevance, no flexible and accessible toolbox currently exists for non-stationary delay adjustment. Here, we present *Nowcast-It*, a tutorial-based toolbox that includes two components: (1) an R code base for delay adjustment and (2) a user-friendly R-Shiny application to enable interactive visualization and reporting delay correction without prior coding expertise. The toolbox supports daily, weekly, or monthly resolution data and enables model performance assessment using metrics such as mean absolute error, mean squared error, and 95% prediction interval coverage. We demonstrate the utility of *Nowcast-It* toolbox using publicly available weekly Ebola case data from the Democratic Republic of Congo. We and others have adjusted for reporting delays in real-time analyses (e.g., Singapore) and produced early COVID-19 forecasts; here, we package those delay adjustment routines into an accessible toolbox. It is designed for researchers, students, and policymakers alike, offering a scalable and accessible solution for addressing reporting delays during outbreaks.

## 1. Introduction

The timely and accurate maintenance of disease surveillance systems helps provide prompt information required to identify risks, predict challenges, allocate public health resources, monitor disease trends, and deploy interventions to contain outbreak situations such as the COVID-19 pandemic [1]. Despite the importance of timely surveillance, delays in reporting case information exist in real time [2], often because the reporting of symptomatic cases is subject to reporting delay, the inherent delay between the time of symptom onset and the time a case is reported in the surveillance system. These reporting delays can vary widely depending on the disease and the health system, ranging from several days (e.g., for influenza and Ebola) to months (e.g., for tuberculosis), and even years in the case of Acute Immuno-Deficiency Syndrome (AIDS). Indeed, reporting delays stem from a mix of biological, behavioral, and operational factors. These include various controllable and uncontrollable processes, such as the incubation period of the infection, delay in case detection, delay in symptom of onset after infection, delay in seeking medical care, delay in diagnostics, delay in processing of surveillance systems, non-contacts, community deaths, and the time required to report the case in the database, etc. [3]. Furthermore, a strong day-of-the-week effect (i.e., very few cases are reported on Saturdays and Sundays) often influences the reporting of cases to surveillance systems [3]. Data quality during an active pandemic and epidemic also undermines reporting delay adjustments with retrospective data backfilling, data corrections, misclassification, and data updates [4]. These epidemiological delays result in a systematic downward bias in recent data, resulting in misleading trends in real-time data used for public health decision-making. The reporting delays are further enhanced in resource-constrained settings, resulting in an increase in the number of new cases by the time of symptom onset, showing a downward bias towards the current day [3,5]. When the reporting delay is minimal, its impact on forecasting models is also small, but when reporting delays are substantial and when real-time estimates and forecasts of disease burden are relied upon to inform policy interventions, additional thought is needed to address their effects [6].

Nowcasting is the process of estimating the actual number of symptomatic-but-not-yet-reported cases in real time, which is essential for correcting this downward bias and enhancing situational awareness during an outbreak. A range of nowcasting models has been proposed to address this challenge. Bayesian smoothing approaches such as NobBS [2] leverage historical patterns and temporal correlation between incidence values to generate probabilistic nowcasts, while constrained P-spline smoothing methods [3] model the reporting intensity surface across onset and delay dimensions. These methods offer valuable flexibility and uncertainty quantification but often assume stationarity in reporting delays and can be computationally intensive or require substantial statistical expertise. In this tutorial paper, we introduce *Nowcast-It*, a user-friendly toolbox based on a frequentist approach rooted in survival analysis and actuarial science. The toolbox estimates reporting delay-adjusted incidence using reverse-time hazard methods and allows for non-stationary delay distributions by specifying a moving-window for delay probability estimation. The underlying delay adjustment routines have been used in previous real-time analyses during outbreaks, including COVID-19 [1,7] and Ebola [5]. The reporting delay-adjusted case incidence estimates can then be utilized to fit and forecast trajectories of infectious diseases using phenomenological dynamic growth models or mechanistic models. This toolbox is written for various audiences, including students training in infectious disease modeling, emergency preparedness and pandemic control, epidemiological time-series forecasting, and dynamic growth modeling. The toolbox is also useful for researchers and policymakers who need to conduct short-term forecasts by relying on historical and real-time trajectory data of the process of interest, such as an unfolding epidemic. We provide R code to assess the performance metrics to choose the best window for non-stationary reporting delays. We have also developed an R-Shiny application to further simplify usage of the toolbox.

Unlike popular statistical nowcasting techniques that principally draw from survival analysis and actuarial methods to model reporting delays, which often assume stationarity (quasi-stationarity) of the reporting delays, our approach uses a flexible, windowed estimation of the delay distribution to accommodate shifts due to changing surveillance systems or field conditions. This makes the toolbox particularly well-suited for low-resource settings, emergencies, or in areas of conflict and political instability where surveillance patterns evolve over time [8]. This robust method employs survival analysis techniques and uses point estimation based on reverse-time hazards [9]. The 95% prediction limits are derived using the statistical estimations introduced by Lawless, modified by assuming non-stationary reporting delay probabilities [10,11,12]. The toolbox includes the creation of line list data, estimation of reporting delay-adjusted trends, variable reporting delay window, and approaches to adjust for reporting delays in situations when data backlogging happens or excessively long reporting delays occur. This survival analysis- and reverse-time hazards-based nowcasting method implemented here is computationally efficient, does not require prior distributions or sampling algorithms, and offers intuitive parameter tuning through the selection of delay estimation windows, making it easy to use.

This tutorial-based primer is organized as follows. After providing an overview of the toolbox functions for users, we first introduce the reporting delay adjustment method included in the toolbox, and then describe the underlying methodology, user parameters, and functions to adjust for the reporting delay. Finally, we use specific examples in the context of the Ebola epidemic in the Democratic Republic of Congo (DRC) to demonstrate the functions for generating, displaying, and quantifying the performance of nowcasts. Application of the *Nowcast-It* toolbox to other data sets is presented in the Supplementary File. A description of the Shiny App’s usage follows towards the end. A tutorial video demonstrating the toolbox functionality is available at the YouTube link: https://www.youtube.com/watch?v=KDYAnXb-IEY&ab_channel=Chowell_Lab (accessed on 28 July 2025).

## 2. Methods

### 2.1. Conceptual Positioning of Nowcast-It

The methodological foundations of *Nowcast-It* predate the emergence of more recent nowcasting frameworks such as NobBS [2] and the constrained P-spline method [3]. The approach implemented here builds directly upon the actuarial and survival analysis formulations introduced by Taylor (1985) [9] and Lawless (1994) [10], which first formalized the statistical treatment of reporting delays in right-truncated surveillance data. These early developments laid the groundwork for subsequent model families that extend the same core principle of reconstructing the true incidence curve from incomplete or delayed reports.

*Nowcast-It* offers a streamlined and computationally efficient realization of these foundational ideas. Rather than competing with the more recent Bayesian or penalized-spline frameworks, *Nowcast-It* provides a transparent frequentist alternative that emphasizes interpretability and accessibility. By employing a closed-form, reverse-time hazard formulation and a simple moving-window parameter (“*m*”) to accommodate non-stationary reporting patterns, the toolbox allows for rapid and stable estimation without the need for prior distributions, iterative sampling, or complex tuning procedures. This design makes it particularly suitable for near-real-time epidemic monitoring, instructional purposes, and deployment in data- or resource-limited contexts where methodological simplicity is a practical advantage. By contrast, NobBS relies on a Bayesian hierarchical structure that enables comprehensive uncertainty quantification through posterior distributions, whereas the constrained P-spline framework models the reporting intensity surface across onset and delay dimensions to yield smooth nowcasts with flexible regularization [2,3].

### 2.2. Implementation

**Installing the toolbox**
  - Download the R code located in the ‘**Reporting delay adjustment code**’ folder from the online GitHub repository: https://github.com/atariq2891 (accessed on 8 August 2025).
  - Create an ‘input’ folder in your working directory where your input data will be stored.
  - Create an ‘output’ folder in your working directory where the output files will be stored.
  - Create a ‘Functions’ folder in your working directory where the function files will be stored.
  - Download and install R-Studio Version 2023.06.1+524.
  - Open an R session so a window called R console appears.
  - Install and run xQuartz (XQuartz-2.8.5.pkg) version 8.2.5 from https://www.xquartz.org/ to visualize the figures produced by the reporting delay adjustment algorithm.

### 2.3. Overview of the Toolbox Functions

Table 1 lists the names of user functions associated with the toolbox along with a brief description of their role.

### 2.4. Data Resolution

The case incidence or mortality data utilized in the nowcasting methodology can be based on a daily, weekly, or monthly resolution, depending on the availability and noise in the data. Data smoothing can be performed to account for the noise in the data [13].

### 2.5. Reporting Delay Adjustment Methodology

**Nowcasting approach**

For this tutorial, we have developed a method to adjust the epidemic curve for reporting delays utilizing the Actuaries’ method suggested initially by Taylor. G in 1986, where it is known as the “claims reserving modeling” method [9]. This method found its application in the biostatistical context analyzing HIV/AIDS data [10,12]. Since then, this method has been widely utilized to understand the reporting delays in the context of various diseases such as Ebola virus disease and COVID-19 [1,14]. We address the nowcasting approach based on the Actuaries’ method in the statistical framework of the occurred-but-not-reported events [10]. Here, the estimation of the delay distribution takes into account the inherent right-truncation of the data-generating process. In general, this method employs survival analysis techniques and uses point estimation based on reverse-time hazards [9]. The 95% prediction limits are derived using the statistical estimations introduced by Lawless, modified by assuming non-stationary reporting delay probabilities [10,11,12]. Daily, weekly, or monthly data can be utilized to ascertain the reporting delays. However, weekly time intervals are mostly used as a compromise between maximizing temporal resolution and reducing reporting irregularities in batch-reported daily counts and inaccuracies in retrospectively ascertained dates of onset.

To explain the approach, we will utilize the weekly Ebola data from the 2018 Ebola epidemic in the DRC. Application of this method to other data sets including COVID-19 data is provided in the Supplementary File [1]. The discrete time-series data published by the week of reporting provide the Ebola case counts by week of symptom onset. In this time-series data, each reported individual is associated with two events: the first event is directly related to the time of illness onset, and the second event is directly related to the diagnosis of the case or the reporting of the case to the surveillance system. Let C denote the “current time,” which is the time point at which data are used for analysis. Let t=0, 1, 2…C denote the time of the occurrence of the event; t=0 is the earliest possible time when an event can occur in the population. Let x=0, 1, 2,…C−t denote the reporting delays, where x=0 indicates that the event is reported in the same time-period when it occurs. Therefore, the data can be grouped as $n_{tx}=\;\#\{initial\;event\;at\;time\;t\;and\;reported\;at\;the\;second\;event\;at\;time\;t+x\},\;where\;x=0,\;1,\;\ldots ,\;C$ − *t* and t=0, 1, …, C.

**Figure 1:**
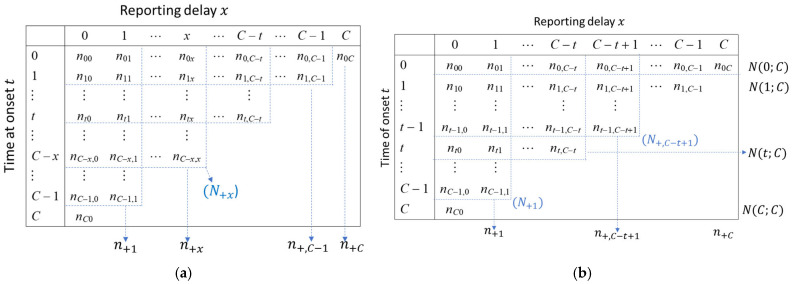
Upper triangular matrix depicting reporting delays. Panel (**a**) describes the matrix with time of onset on the *y*-axis and reporting delay on the *x*-axis and column totals. Panel (**b**) describes the matrix with time of onset on the *y*-axis and reporting delay on the *x*-axis with row and column totals.

As in Figure 1, ${\;N}_{+x}=\sum_{t=0}^{C-x}\sum_{j=0}^{x}n_{tj}=\#\{delay\leq x\}$, and $n_{+x}=\sum_{t=0}^{C-x}n_{tx}=\#\left\{delay=x\right\}\;,\;$ out of the events in t=0, 1,…..,C−x.

This data only fills the upper triangular array of the matrix due to right-truncation, with column totals representing the number of events with delay = X=x and row totals representing the number of events as reported by the current time, C (Figure 1).

The reporting delay adjustments are based on the above-mentioned reverse hazard function and given as $\hat{g_{x}}=\frac{n_{+x}}{N_{+x}}$. We define the maximum likelihood estimate as $\hat{g_{x}}=\frac{n_{+x}}{N_{+x}},$ based on a multinomial likelihood function where delays are denoted by x=1,2, …, C.

This implies that $\hat{g_{x}}=\frac{\#\{delay=x\}}{\#\{delay\leq x\}}$ out of events in t=0,…,C−x. This is the empirical conditional probability ($\hat{Pr}(X=x|X\leq x)$).

Therefore,

(1) $$1-\hat{g_{x}}=1-\frac{\#\{X=x\}}{\#\{X\leq x\}}=\frac{\#\{X\leq x\}}{\#\{X\leq x\}}-\frac{\#\{X=x\}}{\#\{X\leq x\}}=\frac{\#\{X\leq x-1\}}{\#\{X\leq x\}}$$

where x=1,2, …, C. This estimates the proportion of events with delay X≤x−1 out of those with delay X≤x. Thus, 1− $\hat{g_{x}}$ estimates the conditional probability ($\hat{Pr}(X\leq x-1|X\leq x)$).

If x=C−t+1, substituting $x=C-t+1\;into\;Equation\left(1\right)$ gives

(2) $$1-\hat{g_{x}}=1-{\hat{g}}_{C-t+1}=\frac{\#\{X\leq C-t\}}{\#\{X\leq \left(C+1\right)-t\}}$$

This estimates the proportion of events with X≤C−t out of those with delay $X\leq \left(C+1\right)-t\;\approx$ ($\hat{Pr}(X\leq C-t|X\leq \left(C+1\right)-t)$. This means it estimates the proportion of individuals with onset at time t  who would be reported by time C, out of those who would be reported by time C+1.

Using 1 − ${\hat{g}}_{u}$=$\frac{\#\{X\leq u-1\}}{\#\{X\leq u\}}$, we obtain a telescoping product

(3) $$\prod_{u=C-t+1}^{C}[1-{\hat{g}}_{u}]=\prod_{u=C-t+1}^{C}\frac{\#\{X\leq u-1\}}{\#\{X\leq u\}}=\frac{\#\{X\leq C-t\}}{\#\{X\leq C\}}\approx \hat{Pr}(X\leq C-t|X\leq C)$$

$$\prod_{u=C-t+1}^{C-t+2}\left[1-\hat{g_{u}\;}\right]=[1-{\hat{g}}_{C-t+1}][1-{\hat{g}}_{C-t+2}]$$

This estimates the proportion of individuals with time onset at time *t* reported by time *C*, out of those who would be reported by time *C* + 2.

Let N(t;C) be the number of cases with onset at time *t* reported by time *C*, and $i\left(t\right)$ defines the true number of onsets at time *t*. Only a fraction $\hat{Pr}(X\leq C-t|X\leq C)$ is observed by time *C*. Thus,

(4) $$N\left(t;C\right)=i\left(t\right)\hat{Pr}\left(X\leq C-t|X\leq C\right)$$

Solving (4) for $i\left(t\right)$ and replacing probability with the empirical estimate from (3) we obtain the reporting delay adjustment factor assuming quasi-stationarity of the reporting delays patterns, and it can be described as follows:

(5) $$\hat{i\left(t\right)}=\frac{N\left(t;C\right)}{\prod_{u=C-t+1}^{C}\left[1-\hat{g_{u}}\right]}=\frac{N(t;C)}{\hat{Pr}(X\leq C-t|X\leq C)},t=1,\ldots ,C.$$

where $\prod_{u=C-t+1}^{C}\left[1-\hat{g_{u}}\right]$ estimates the proportion of individuals with time onset at time t reported by time C out of those would be reported by time C+1 and $\hat{i\left(t\right)}$ is the reporting delay adjustment. Therefore, $N\left(t;C\right)=i\left(t\right)*Pr(X\leq C-t|X\leq C)$.

Assuming stationarity across the reporting delay patterns yields less robust estimates of delay adjustment, we use a modified approach in which we choose the most recent reporting periods (assuming relatively stable reporting delay patterns in the most recent reporting time periods), owing to irregular reporting delay patterns captured by surveillance systems over longer reporting periods.

### 2.6. Assuming Non-Stationarity in the Reporting Delays

While some reporting delay distributions are expected to remain stationary during an epidemic wave, others can change over time in response to various factors including interventions and retrospective data corrections [15,16]. Since reporting delays are not stationary in the real world, we employ a modified approach by selecting a variable reporting delay window, defined as “*m*”, to address this issue of stationarity. Disease reporting patterns are generally influenced by external factors (i.e., delay in disease detection, late diagnostic test and healthcare system report, suspected versus confirmed cases, and hospitalization intake) and are unstable [6,17]. In most countries, national or local reporting systems produce regular reports providing the most up-to-date statistics on disease diagnoses. However, these provisional reports may be revised/corrected through a process called backfilling or backlogging as new data becomes available. Therefore, real-time reports of current disease burden often substantially underestimate the number of cases eventually reported for a given time period [2,6]. During epidemic and pandemic situation, disease reporting patterns in surveillance systems are ever-changing. Therefore, it is more likely for the reporting patterns to be similar (stationary) across few weeks or days than across the entirety of the epidemic period.

We observe a systematic departure from stationarity of the reporting delays in the data used for the tutorial, which can be explained by changes in reporting, data misclassification, retrospective data corrections, and observable events like the escalation of conflict during the Ebola epidemic in the DRC. Hence, we treat reporting patterns to be stable during the different smaller reporting periods, such as during the last 6–8 weeks, implying stationarity during those periods only. The window “*m*” represents the trends of the reporting delay probabilities for the chosen time period, assuming stable reporting delay patterns only in that time period. We use different values of “*m*” and compare the performance of different windows “*m*’s” across the data sets, each set apart by one week. The “*m*’s” employed in this study include the most recent reporting periods (7, 9, and 11 weeks, and 3, 6, and 8 weeks), along with the best “*m*” value. To choose the best “*m*” value, we plot the ratio of delays that occur in the same week (delay0) to the delays that occur in the first week of onset (delay1) (ratio delay0/delay1). This provides us with the calculated probability for the last prediction and helps in choosing the best “*m*” value. This one-step prediction can be quantified as follows:

$$\hat{N}\left(t;C+1\right)=\frac{N\left(t;C\right)}{1-{\hat{g}}_{C-t+1}}=N\left(t;C\right)\frac{N_{+,C-t+1}}{N_{+,C-t+1}-n_{+,C-t+1}}$$

However, it can only be performed for t=1, 2,…,C. In this equation, $1-{\hat{g}}_{C-t+1}$ estimates the proportion of individuals with onset at time t  reported by time C, out of those who would be reported by time $C_{1}=C+1$. The performance of this one-step prediction (also known as the partial delay adjustment) also informs us about the performance of our ultimate reporting delay adjustment. Sometimes we use the reporting delay distribution pattern for the entire data set (assuming a stationary reporting delay distribution across the entire data set) to compare the differences between choosing stable versus variable reporting delay patterns that are best suited for a data set to achieve the most robust adjustment of the raw incidence.

### 2.7. Evaluating the Performance of Nowcasts, Performance Metrics

To assess the performance of our nowcasting method using differing values of “*m*”, we employ three performance metrics: mean absolute error (MAE), mean square error (MSE), and the 95% prediction interval. The MAE is given by

$$MAE=\frac{1}{N}\sum_{i=1}^{N}\left|Adjusted\;Incidence(i)-True\;Incidence(i)\right|$$

and the MSE is given by

$$MSE=\frac{1}{N}\sum_{i=1}^{N}(Adjusted\;Incidence(i)-True\;Incidence(i){)}^{2}$$

In addition, we assessed the coverage of the 95% prediction interval, the proportion of the observations that fell within the 95% prediction interval. The 95% prediction coverage is given as follows:

$$PI\;coverage=\;100\times (sum(data\;(i)\leq UB\left(i\right)\;and\;data(i)\;\geq LB\;(i)))/length\;(data)$$

where UB = upper bound and LB = lower bound.

In this tutorial example, the performance metrics are estimated against the true incidence reported eight weeks later (n=8) for the same dates of onset. True incidence varies for different epidemics (two weeks later, eight weeks later, four months later, etc) and needs to be identified based on the reporting practices and the time when the case counts have become complete. All performance metrics for the nowcasting approach are computed in R-Studio Version 2023.06.1+524.

## 3. Results and Discussion

### 3.1. The Input Data Set

For this toolbox, the time-series data must be stored as line-listed data in the ‘input’ folder and needs to be a text file with the extension *.txt. Data can be stored in the form of case or death count data. For this tutorial, we are using case count data. Each case should be a separate row in the data set, such that the first column should correspond to the date/week of symptom onset (1, 2, 3, …), and the second column corresponds to the date/week of reporting (1, 2, 3…). If you are using the dates of onset and reporting, the five-digit number representing the date of onset, counted from 30 April 2018, and the date of reporting, 20 August 2018, is created by selecting two fields originally entered in date format and converting them to number format in Excel. For example, 20 August 2018 is converted to 43,332.00. This Excel file should then be saved as a *.txt* file.

**Figure 2:**
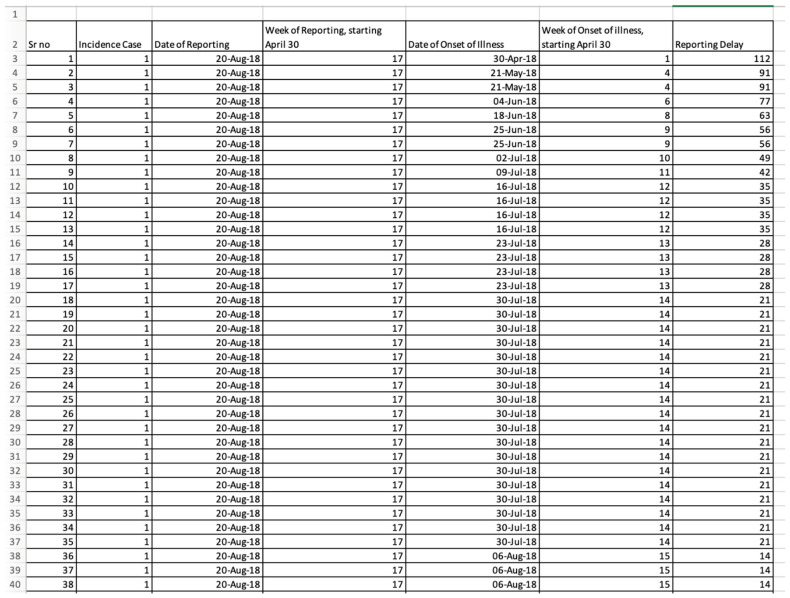
Snapshot of the Excel data file with dates created from the WHO situation report published on 3 March 2019.

**Figure 3:**
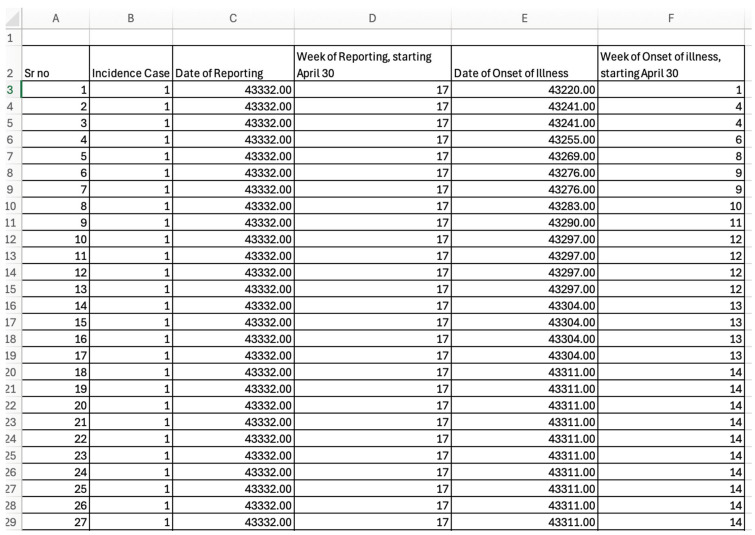
Snapshot of the Excel data file with dates converted to number format, created from the WHO situation report published on 3 March 2019.

**Figure 4:**
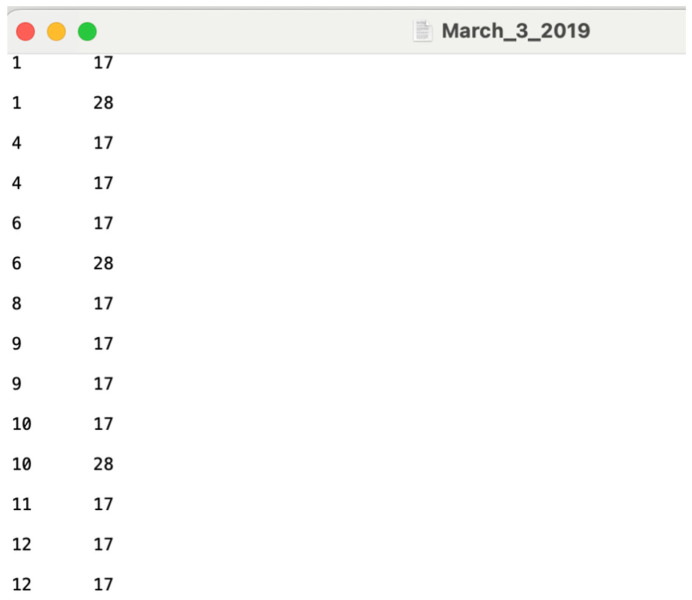
Snapshot of the *.txt* file saved with weeks of onset and reporting in the number format for the Excel file dated on 3 March 2019. Data is stored at a weekly resolution. The first column represents the week of symptom onset, and the second column represents the week of reporting of the Ebola cases.

**Figure 5:**
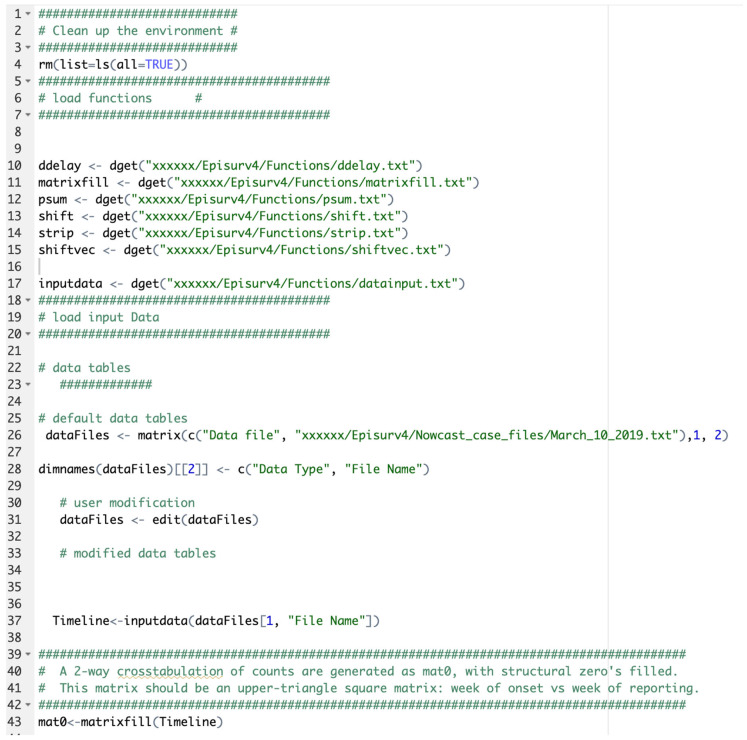
Components of the *load-data-and-function.r* file. Insert the pathway to the files and functions where “xxxxxxx” is indicated.

To illustrate the methodology presented in this tutorial paper, we employ the weekly incidence curve of the Ebola epidemic in the DRC. Data are extracted from the situation reports publicly available from the World Health Organization website [18]. The data file is located in the input folder within the working directory (data file path: ./input/March_3_2019.*txt*). A snapshot in Excel (Figure 2 and Figure 3) and *.txt* (Figure 4) of the contents of the file is shown below:

The *load-data-and-functions.R* file is an R-Studio program that allows users to load all functions into the algorithm (Figure 5). This file contains the algorithm to produce a two-way crosstabulation of case/death counts that are generated as “mat0”, as structural zeros fill in the matrix. This matrix is a square upper-right triangle matrix representing the week of onset versus the week of reporting. The algorithm also produces an upper-left triangular square matrix, “mat”, representing the week of onset versus the week of reporting. This matrix is utilized in the “ddelay” function.

**Figure 6:**
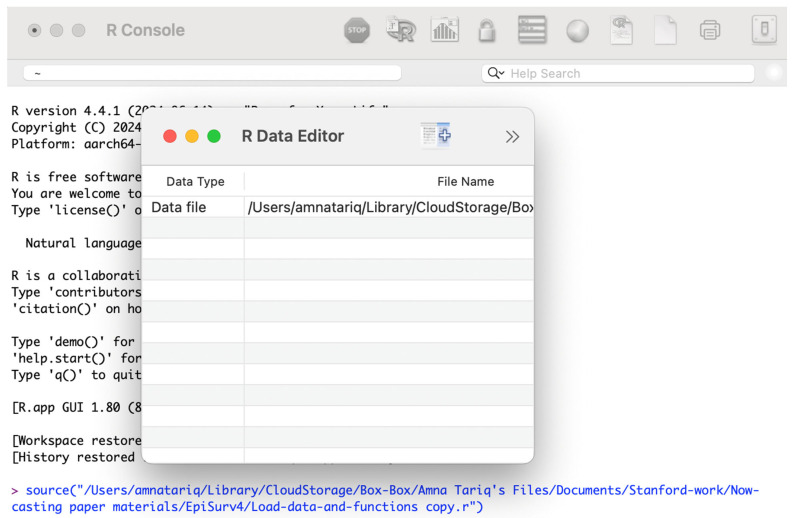
Dialog box as seen using the code Source (“c:/EpiSurv/load-data-and-functions.r”) in R console.

In order to run the code to estimate the adjusted case incidence, open R console and type *Source (“c:/EpiSurv/load-data-and-functions.r”).* This will open a dialog box. For the current data, type the input filename as March_3_2019.*txt*. Then close the dialog box (Figure 6).

**Figure 7:**
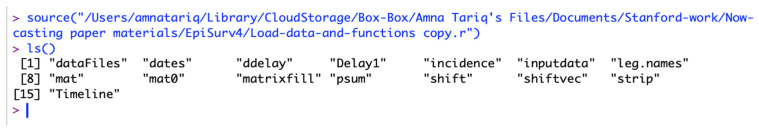
List of elements in ls().

In order to check the contents of the file, type ls(). It will produce a list of the functions and items that have been created. The following results are produced (Figure 7).

### 3.2. Results

**Figure 8:**
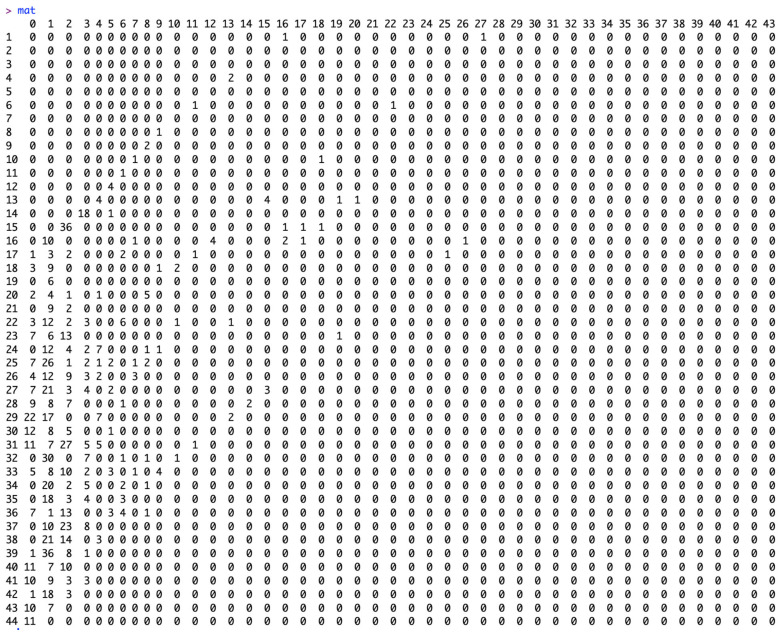
Elements of the matrix “mat” which is an upper-left triangular square matrix with rows representing the week of onset and columns representing the reporting delay.

**Figure 9:**
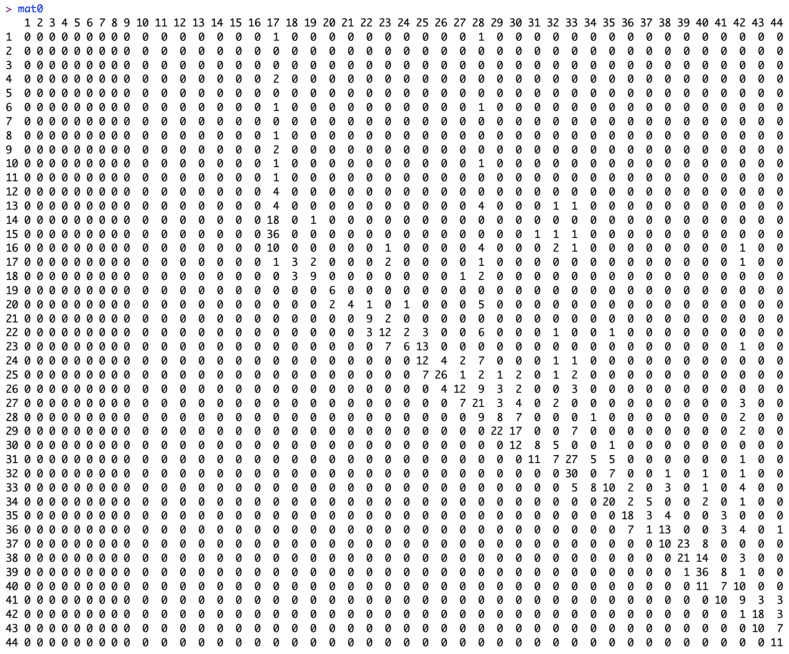
Elements of matrix “mat0” which is an upper-right triangular square matrix with rows representing the week of onset and columns representing the week of reporting.

To check the components of the right and left upper triangular matrices, we provide the command “mat” and “mat0”. The matrix “mat” is an upper-left triangular square matrix with rows representing the week of onset and columns representing the reporting delay (Figure 8). The matrix “mat0” is an upper-right triangular square matrix with rows representing the week of onset and columns representing the week of reporting (Figure 9).

**Figure 10:**
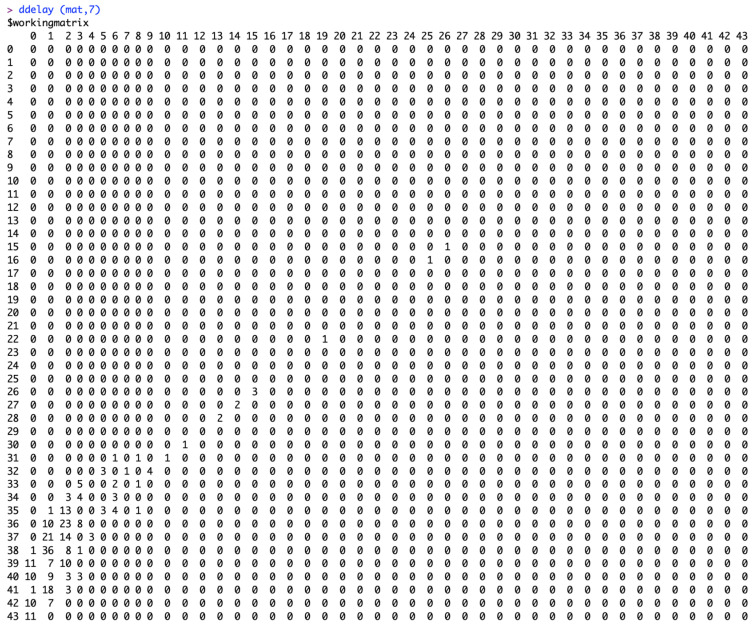
Elements of matrix “mat” with a non-stationary reporting delay window *m* = 7.

**Figure 11:**
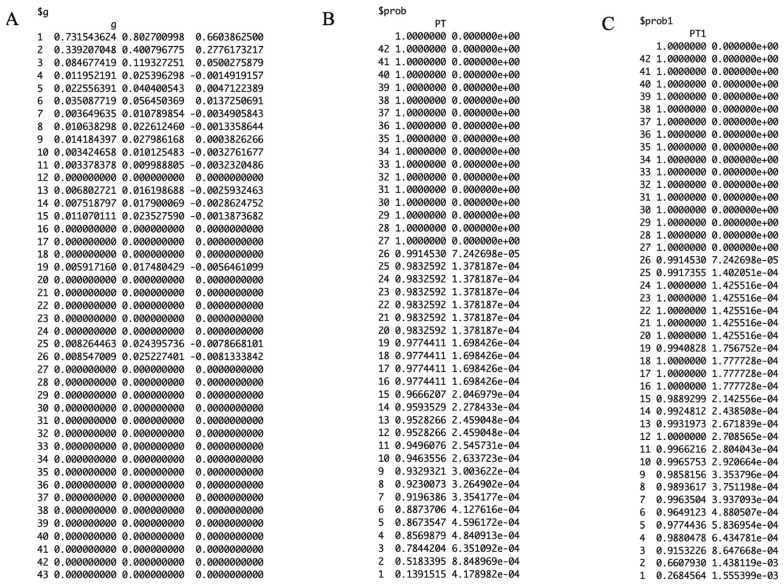
Probabilities for the data set generating the reporting delay-adjusted incidence. Panel (**A**) “$g” represents the estimated reverse hazards with the confidence intervals, Panel (**B**) “$prob” and panel (**C**) “$prob1” represents, the right-truncated conditional probabilities as the delay adjustment factor.

**Figure 12:**
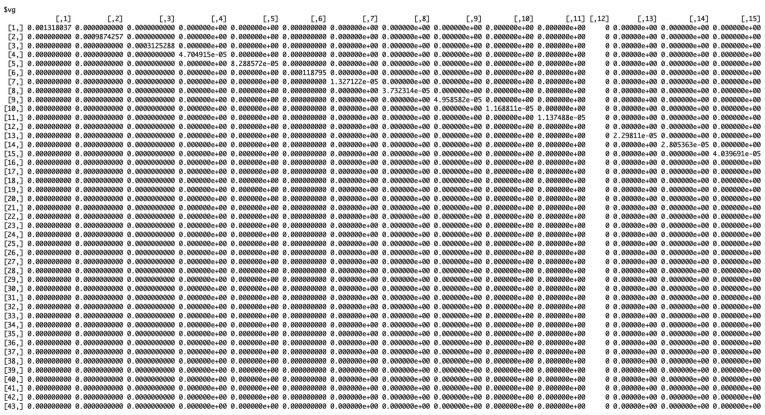
Matrix for probabilities for the week of onset versus the week of reporting.

**Figure 13:**
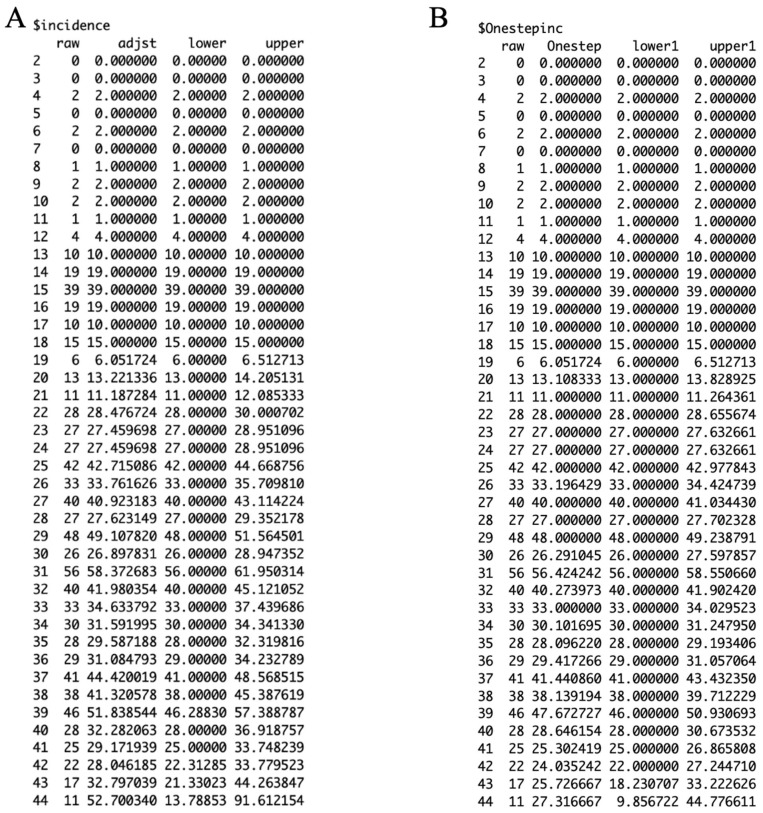
Panel (**A**) presented as “$incidence” contains the reporting delay-adjusted incidence output with the first column representing the dates, the second column representing the raw incidence data, the third column representing the adjusted incidence, and the fourth and fifth columns representing the lower and upper adjusted incidence respectively. Panel (**B**) presented as “$Onestepinc” provides the one-step prediction estimates, with the first column representing the dates, the second column representing the raw incidence data, the third column representing the one-step predicted adjusted incidence, and the fourth and fifth columns representing the one-step predicted lower and upper adjusted incidence respectively.

**Figure 14:**
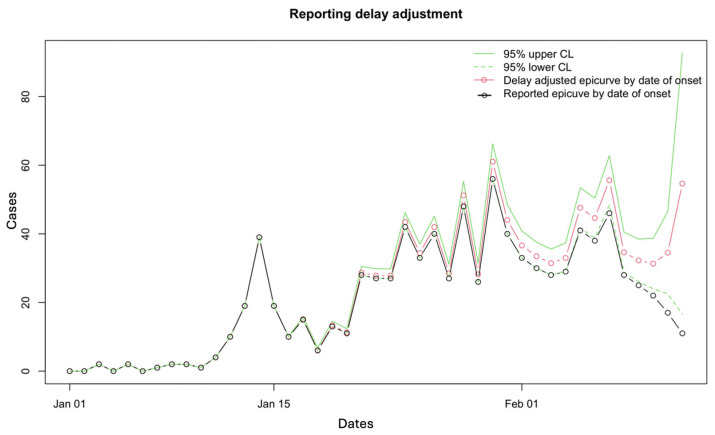
Reported delay-adjusted incidence curve. The dashed green lines represent the lower 95% confidence limit, and the solid green lines represent the upper 95% confidence limit. The red line and open circles represent the delay-adjusted epidemiologic curve by date of onset, and the black solid line and open circles represent the reported epidemiologic curve by date of onset.

The function “ddelay” contains the algorithms from Lawless (1994) [10]. The input data for the “ddelay” function is the matrix “mat”. For the “ddelay” function, there is a choice to provide the value of “*m*”. This allows us to select the most recent reporting period “*m*” (according to the week of reporting) that we believe is the time during which the reporting practice has been reasonably stable. If we set *m* = ncol(mat), then the algorithm assumes a stable reporting delay distribution from the beginning of the data set, which is not true in most instances. The command line to produce the required output is ***ddelay(mat, m).*** Figure 10 shows the working matrix when *m* = 7. The list of outputs it produces includes the estimated reverse hazard with confidence limits, the right-truncated conditional probabilities as the delay adjustment factor, and a table of reporting delay-adjusted incidence (Figure 11, Figure 12 and Figure 13). Figure 14 shows the curve of the reporting delay-adjusted incidence.

### 3.3. Performance Metrics

**Figure 15:**
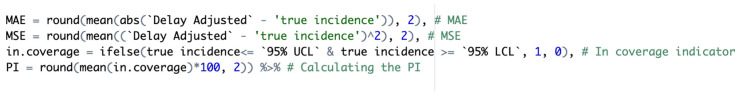
Algorithm to estimate the MAE (mean absolute error), MSE (mean squared error), and the 95% prediction interval (PI) coverage.

We assess the performance metrics of the different nowcasts by varying the value of “*m*” and comparing the difference from the true incidence eight weeks later (Figure 15). We utilize the reporting delay-adjusted incidence, true incidence, upper limit of the reporting delay-adjusted incidence, and the lower limit of the reporting delay-adjusted incidence from the output file (Figure 13A) to estimate the performance metrics (Table 2).

**Figure 16:**
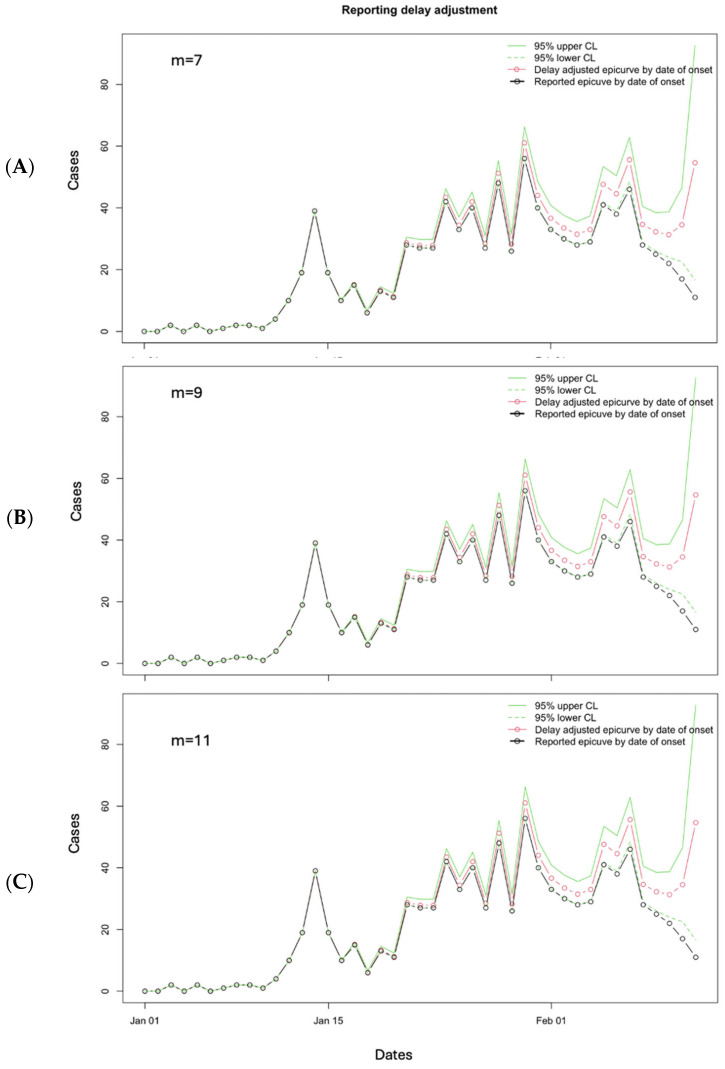
Reporting delay adjustment curves for the Ebola epidemic as reported on 3 March 2019, adjusted according to *m* = 7 (**A**), *m* = 9 (**B**), and *m* = 11 (**C**). The dashed green lines represent the lower 95% confidence limit, and the solid green lines represent the upper 95% confidence limit. The red line and open circles represent the delay-adjusted epidemiologic curve by date of onset, and the black solid line and open circles represent the reported epidemiologic curve by date of onset.

Based on the results of the performance metrics, the best “*m*” value would be *m* = 7 for 3 March 2019, assuming a non-stationary reporting delay pattern of seven weeks. Below, we show the panel figure representing the reporting delay-adjusted incidence curve using different values of “*m*” for the 3 March 2019 data file (Figure 16).

**Figure 17:**
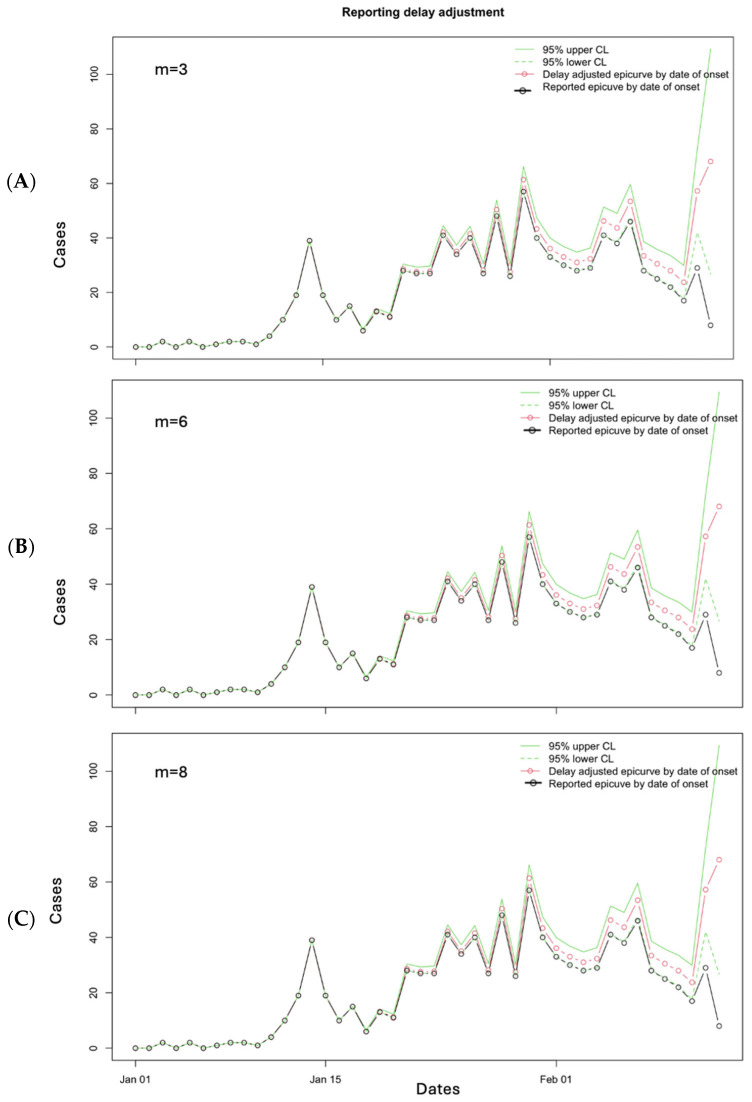
Reporting delay adjustment curves for the Ebola epidemic as reported on 10 March 2019, adjusted according to *m* = 3 (**A**), *m* = 6 (**B**), and *m* = 8 (**C**). The dashed green lines represent the lower 95% confidence limit, and the solid green lines represent the upper 95% confidence limit. The red line and open circles represent the delay-adjusted epidemiologic curve by date of onset, and the black solid line and open circles represent the reported epidemiologic curve by date of onset.

Similarly, when we use the data file for 10 March 2019 and “*m*” values of 3, 6, and 8, we obtain the following figure (Figure 17), and the performance metrics are given in Table 2. The window “*m*” = 3 provides the best results for MSE (12.6) and the window of “*m*” = 8 provides the lowest value of MAE (1.85). The 95% PI coverage is the same for all three values of “*m*”.

**Figure 18:**
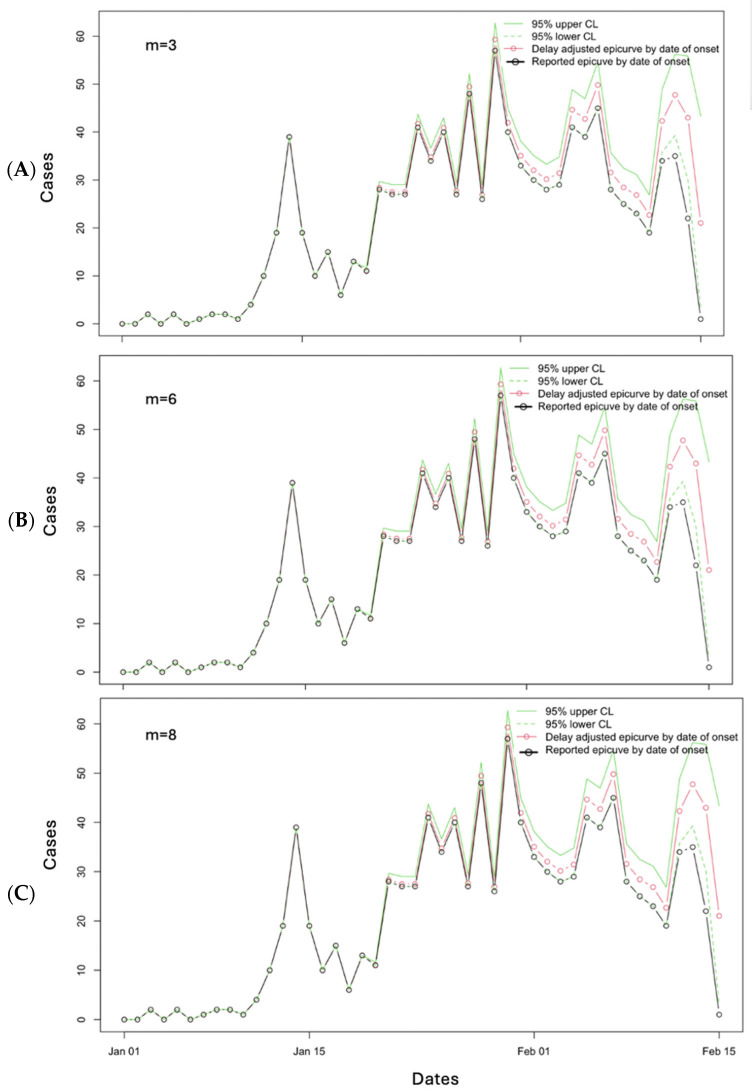
Reporting delay adjustment curves for the Ebola epidemic as reported on 19 March 2019, adjusted according to *m* = 3 (**A**), *m* = 6 (**B**), and *m* = 8 (**C**). The dashed green lines represent the lower 95% confidence limit, and the solid green lines represent the upper 95% confidence limit. The red line and open circles represent the delay-adjusted epidemiologic curve by date of onset, and the black solid line and open circles represent the reported epidemiologic curve by date of onset.

When we use the different values of “*m*” (3, 6, and 8) for 19 March 2019, the following figure can be produced (Figure 18). The performance metrics are given in Table 2. Based on the results of the performance metrics, the window of “*m*” = 3 provides the best results for the MAE (2.17) and “*m*” = 8 provides the best results for MSE (69.68) and the 95% PI coverage (91.30).

### 3.4. Shiny App in R

**Figure 19:**
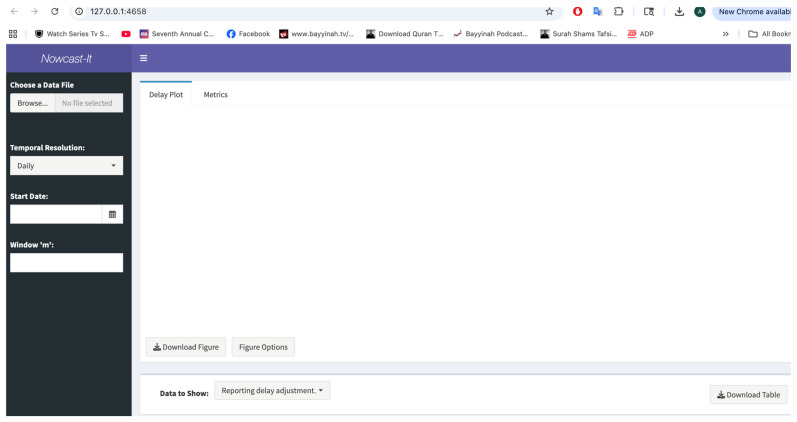
A screenshot of the *Nowcast-It R-Shiny* App (v1.0.0; R 4.3; R-Studio 2024.09.0+375) user interface. This figure provides a snapshot of the user interface for obtaining the reporting delay adjustment curves and other associated data files. As can be seen, the user has options to choose a data file, and then must specify information related to the temporal resolution of the data and the length of the reporting delay window, *m*.

Often, those training in public health or policymakers lack the programming expertise to quickly implement tools that can provide critical information for forecasting efforts, such as reporting delay-adjusted curves. Therefore, we provide an R-Shiny App interface to facilitate the obtainment of reporting delay adjustment curves without requiring any previous programming or coding experience (Figure 19).

The Shiny App interface utilizes the functionality discussed above but provides a graphical interface for users to input their data, specify the temporal resolution, and indicate the number of most recent time points (according to the reporting date) in which we believe the reporting practice has been reasonably stable (*m*). Additionally, users can customize and download the reporting delay adjustment curves, such as those shown in Figure 14, obtain the data from Figure 8, Figure 9, Figure 10, Figure 11, Figure 12 and Figure 13 as ‘.*csv*’ files, and assess the reporting delay adjustment against the “truth” data. The following sections describe the implementation of the dashboard and provide additional examples of the output produced.

### 3.5. Installing and Loading the Shiny App

As above, the Shiny App requires both R and R-Studio for compilation. However, the implementation steps differ slightly:

- Download the ***Nowcast-It Shiny*** folder containing the app and required functions from https://github.com/atariq2891/Reporting-delay-adjustment-code/tree/main (accessed on 27 July 2025).
- Load the ***Nowcast-It Shiny*** project file in R-Studio.
- Open the ‘app.R’ file and click ‘Run App’.

The app requires multiple packages to function; however, they should be downloaded automatically upon the first rendering of the shiny application. Table 3 provides additional details regarding compilation requirements, as well as the web address for the host repository.

**Figure 20:**
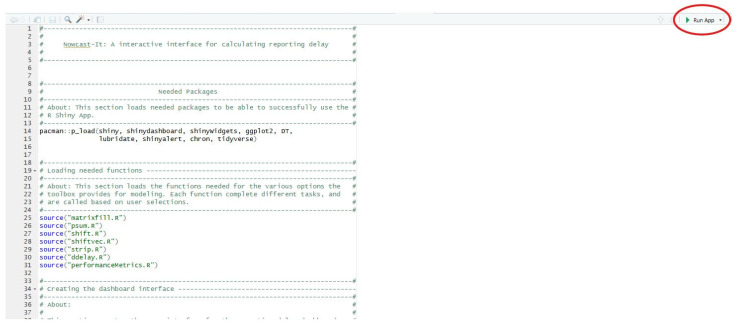
R Project app that launches the Nowcast-It R-Shiny Application. The ‘Run App’ button launches the Nowcast-It application on the user’s local machine.

Prior to launching the R-Shiny application, the user first must load the R Project file located within the downloaded GitHub repository. Launching the R Project prior to running the R-Shiny ensures that the working directory is properly set up on the user’s local machine, and all functions and folders are correctly referenced. Once loaded, the users can then open the ‘app.R’ file via the panel in the bottom-right portion of the R screen. Finally, the user should hit the ‘Run App’ button on the top-right corner to launch the *Nowcast-It* application on their local machine (Figure 20).

### 3.6. Inputting the Data

The dashboard utilizes the same data format presented in Figure 4 and does not require any specific naming scheme. To load data into the dashboard, users can select the *Browse* button located under the “Choose a Data File” header and identify the file location within their personal computer. Once loaded, the user can then specify parameters related to the data’s temporal resolution and for calculating the adjusted incidence curve (Figure 19).

### 3.7. User Specifications

Once data has been loaded, the users can then specify the temporal resolution of their data (i.e., *Temporal Resolution*). The dashboard works with continuous incident or mortality data for daily, weekly, monthly, and yearly periods. For each possible temporal resolution, a calendar will be available under *Start Date*, where users can select the first date (i.e., day, start of week, month, or year) of the available data. Only one date can be selected at a time. Users also have the option to adjust the value of *m*. When initially loading the data, the *m* parameter will default to the maximum number of time points available.

### 3.8. Available Output

**Figure 21:**
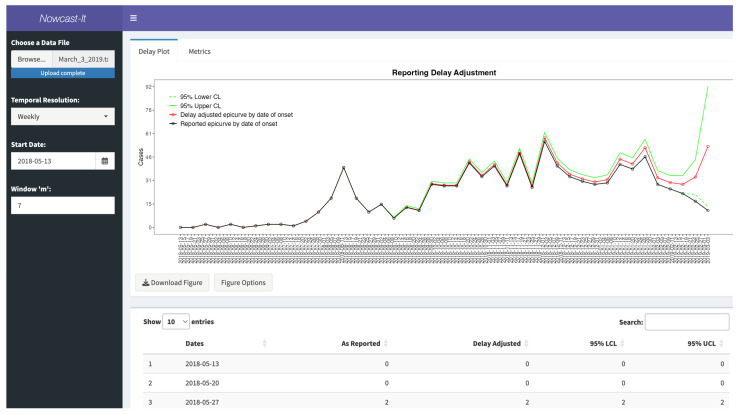
The reporting delay adjustment curve was obtained from the Nowcast-It dashboard. This figure shows the reporting delay adjustment curve obtained from the Nowcast-It dashboard using weekly Ebola data through 3 May 2019 and setting *m* = 7. The dashed green lines represent the lower 95% confidence limit, and the solid green lines represent the upper 95% confidence limit. The red line represents the delay-adjusted epidemiologic curve by date of onset, and the black solid line and open circles represent the reported epidemiologic curve by date of onset.

Once data has been loaded and a *Start Date* selected, the dashboard will auto-populate with the reporting delay adjustment curve (Figure 21), and the following multiple data sets: (1) the reporting delay adjustment, (2) one-step ahead prediction of data, (3) estimated reverse hazards with CI limits, (4) the right-truncated probabilities, and (5) the matrix for probabilities.

The figure is fully customizable by clicking the *Figure Options* button and can be downloaded as a variety of file types. The data are available to download as *csv files. When the underlying data, date options, or parameter *m* is changed, the files will update to reflect the new settings.

### 3.9. Performance Metrics

**Figure 22:**
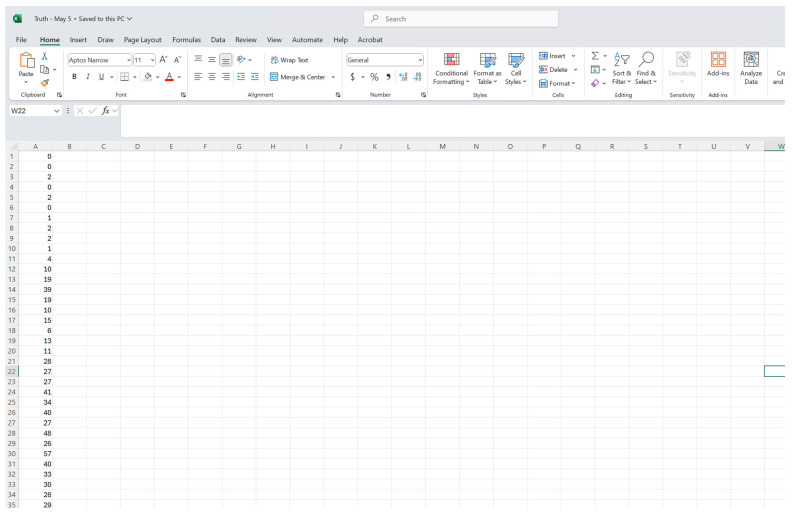
A screenshot of the required data format for the “truth” incidence data. The truth incidence data, used as part of the performance metrics calculations, must be a *txt or *csv file and contain one column corresponding to the true incidence at each time point. The first row of the data must correspond to the first time point available in the delay-adjusted incidence curve. There is no required naming scheme for the file.

As discussed above, it is possible to assess the delay-adjusted incidence curve against data that are believed to represent the true incidence of disease over time. Once the reporting delay-adjusted curve has been calculated, the dashboard provides the mean absolute error (MAE), mean squared error (MSE), and 95% prediction interval coverage for download if the user provides the “truth” incidence data. The truth incidence data must be in either a *.txt* or *.csv* file format, containing only one column of counts over the time of interest and no column headers (Figure 22). The first row of the file must correspond to the first time point of data available in the delay-adjusted incidence curve. There is no required file naming scheme.

The dashboard employs the same MAE, MSE, and 95% PI metrics provided above to ensure robust use with varying data types and lengths. Users can download the resulting metrics as a *csv to their personal computer. Results of performance metrics are given in Table 4.

### 3.10. Limitations to Nowcasting Approach and How to Handle Them

During real-time data surveillance in epidemic and pandemic situations, issues such as outlier data points, excessively long reporting delays, or data corrections and data backlogging can result in inaccurate nowcasts. To address these issues, users need to understand the data and employ approaches to exclude or manage the weeks/days when data backlogging occurs or excessively long reporting delays are observed. Examples of such issues and methods to address them are described below.

**Figure 23:**
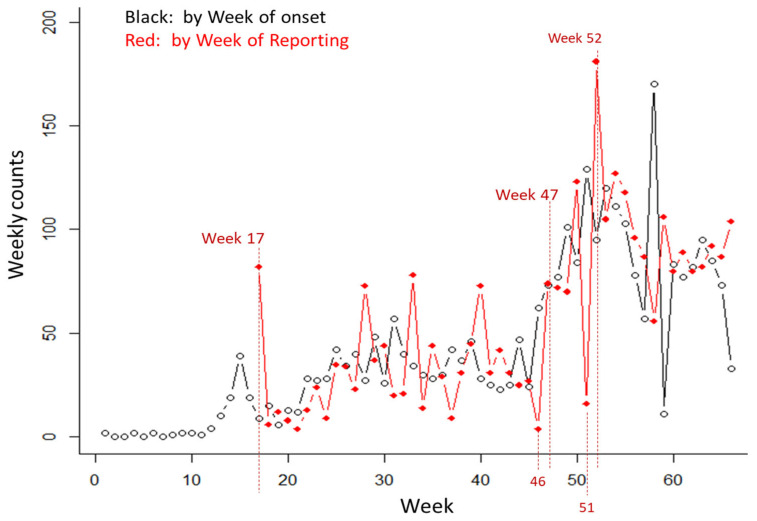
Depiction of the long reporting delays as reported in the situation report published on 9 August 2019. The black line with open circles indicates the incidence curve by week of onset and the red line with filled circles is the incidence curve by the week of reporting.

In the case of the Ebola epidemic in the DRC, we encountered two issues. First, there was an unusually long delay that suddenly emerged as an outlier as reported in the 9 August 2019 report. This long delay occurred possibly due to a case review effort in one of the local reporting jurisdictions. Among the 104 cases reported during the most recent week (week 66), 33 cases were associated with onsets in the same week; 52 cases with onsets in the previous week; and 11 cases with onsets in Week 64. There were four cases with onsets in week 63, and seven cases with long delays, including one case with onset in week 21 but reported in week 66 (reporting delay = 45 weeks). The result was a very rough pattern in the non-parametric estimate of the reverse hazard function, along with overly estimated reporting delay adjustment. Normally, it would take a long time for the data to accumulate to determine whether this was just an outlier or a change in the reporting delay pattern (Figure 23). This can be corrected by considering these data points with delays of 45 weeks as an outlier and excluding them from the data set.

**Figure 24:**
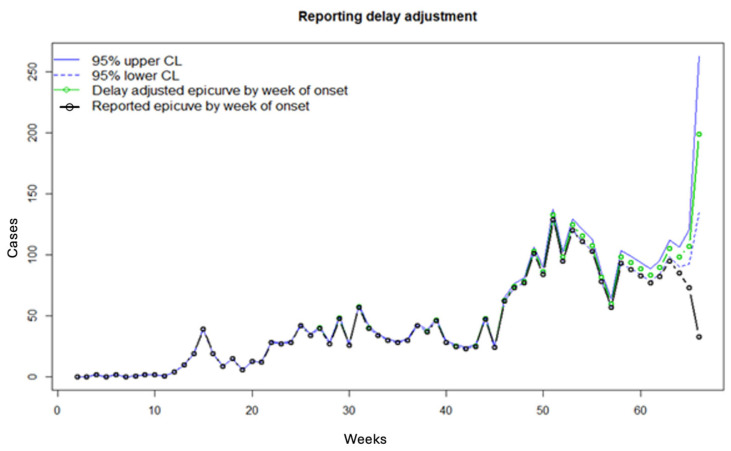
Sharp reporting delay adjustment in weeks 66 and 67 due to an increase in cases (33 to 67 cases) from week 66 to week 67 using the value of *m* = 66 (entire data set). The dashed blue lines represent the lower 95% confidence limit, and the solid blue lines represent the upper 95% confidence limit. The green line and open circles represent the delay-adjusted epidemiologic curve by date of onset, and the black solid line and open circles represent the reported epidemiologic curve by date of onset.

Secondly, in the situation report published on 16 August 2019, the number of Ebola cases increased from 33 cases (symptom onset week 66) to 67 cases (symptom onset week 67). This provided us with a very large value in the last reported case (that is $n_{C0}$), and the number of events in the current time point with delay = 0. This resulted in a sharp reporting delay-adjusted trend that cannot be easily deciphered by just looking at the data. There is also no way of knowing if there was a sharp rise in the disease in the community or a sudden ramp-up of case reporting in the system. This kind of error can be corrected by excluding the weeks before week 58 or by replacing the last data point in week 66 and week 67 as the running median of the previous three data points (Figure 24).

## 4. Conclusions

This tutorial presents a practical nowcasting toolbox, *Nowcast-It*, designed to adjust for reporting delays in real time using a flexible frequentist approach. In particular, the method described in the toolbox has been frequently applied to nowcast the epidemic trajectories in near real time [1,5,7]. The toolbox can be used as part of the curriculum for student training in mathematical biology, applied statistics, infectious disease modeling, and specialty courses in epidemic modeling and time-series nowcasting and forecasting. The data created via this approach can then be utilized to forecast the trajectory of the ongoing epidemics accurately. It is also a helpful resource for researchers and policymakers who can acquire better estimates of missing and unreported cases that can be used to conduct short-term forecasts by relying on historical and real-time trajectory data of a process of interest.

The survival-based method implemented here is computationally efficient, does not require prior distributions or sampling algorithms, and offers intuitive parameter tuning through the selection of delay estimation windows. This makes it accessible for public health practitioners and researchers without deep expertise in Bayesian statistics or advanced computational methods. Moreover, the *Nowcast-It* toolbox includes built-in tools to evaluate model performance using metrics such as mean squared error, coverage probability, and prediction interval width—important features for evaluating forecast reliability. The application of the toolbox to the Ebola epidemic and the COVID-19 data (in Supplementary File) has demonstrated that this framework has the desired flexibility and complexity. The implementation of the toolbox in frequentist framework is extremely fast as indicated from the application of the code in R and the Shiny app.

Despite its strengths, the toolbox has several limitations. First, it relies on the line list data, which may not always be available or complete, particularly in low-surveillance contexts. Second, while the toolbox provides point estimates and confidence intervals based on Lawless’ method, it does not offer full posterior distributions or simulation-based prediction intervals that are available in Bayesian approaches. The method is also sensitive to changes in recent reporting process. The method emphasizes reporting delay distribution, but it does not fully utilize the underlying autocorrelated disease trend driven by transmission. In conclusion, *Nowcast-It* provides a practical and interpretable framework for correcting real-time reporting delays in infectious disease surveillance. Its combination of methodological rigor, computational efficiency, and accessibility makes it a valuable resource for epidemic response and training in applied infectious disease modeling.

## Figures and Tables

**Table 1:** Description of user functions associated with the toolbox.

Function	Type	Role
datainpuut.*txt*	User	Reads a data file as input matrix.
ddelay.*txt*	User	Contains the algorithms of lawless based on reverse-time hazards and survival analysis. The input data is the matrix “mat”. There is a second choice, called “*m*”. This is to select the most recent “*m*” weeks (according to week of reporting) that we believe the reporting practice has been reasonably stable.
delay1.*txt*		Creates a temporary working matrix to be used in ddelay.*txt* function.
matrixfill.*txt*	User	Creates the matrices for the right-truncated data.
psum.*txt*	User	Adds the components of the matrices.
shift.*txt*	User	Shifts the vector to the right or left.
shiftvec.*txt*	User	Creates the time-lagged matrix. Shifts the vector to the right or left.
strip.*txt*	User	If else function for the matrices.

**Table 2:** Results of performance metrics for selecting the best “*m*” from the 2019 Ebola epidemic in the DRC. MSE represents the mean squared error, MAE represents the mean absolute error, and 95% PI represents the 95% prediction interval coverage. Best value is presented in bold. The values of “*m*” can be varied according to the user. Any value of “*m*” can be applied and checked for its robustness using the performance metrics.

Date of Situation Report	MAE	MSE	95% PI Coverage
3 March, *m* = 7	**1.66**	**15.13**	**95.34**
3 March, *m* = 9	1.87	30.49	**95.34**
3 March, *m* = 11	2.26	46.56	**95.34**
10 March, *m* = 3	2.11	**12.6**	**95.45**
10 March, *m* = 6	1.98	30.8	**95.45**
10 March, *m* = 8	**1.85**	29.9	**95.45**
19 March, *m* = 3	**2.17**	90.02	86.9
19 March, *m* = 6	2.76	91.29	**91.3**
19 March, *m* = 8	2.37	**69.68**	**91.3**

**Table 3:** Nowcast-It metadata. Below includes details regarding the required software, versions, and packages needed to launch the Nowcast-It R-Shiny application successfully. Additionally, it provides a link to the permanent repository, which contains all necessary functions, user interface files, and tutorial materials.

Required Software	R (≥4.3), R-Studio (2024.09.0 Build 375)
**Compilation requirements ^1^**	*pacman, shiny, shinydashboard, shinyWidgets, ggplot2, DT, lubridate, shinyalert, chron*
**Permanent link to repository**	https://github.com/atariq2891/Reporting-delay-adjustment-code/tree/main/Shinyapp (accessed on 27 July 2025).

^1^ upon initial compiling of the dashboard, it checks if the required packages are downloaded. If they are not, R will proceed to install the packages for the given session. During this process, pop-up messages may appear asking if the user would like to compile the package. To successfully utilize all features of the dashboard, the user must select “yes”.

**Table 4:** Results of performance metrics from the Shiny app. MSE represents the mean squared error, MAE represents the mean absolute error, and 95% PI represents the 95% prediction interval coverage. Best value is presented in bold. The values of “*m*” can be varied according to the user. Any value of “*m*” can be applied and checked for its robustness using the performance metrics.

	MAE	MSE	95% PI Coverage
3 March, *m* = 7	**1.66**	**15.13**	**95.35**
3 March, *m* = 9	1.88	30.5	**95.35**
3 March, *m* = 11	2.27	46.56	**95.35**
10 March, *m* = 3	2.11	**12.64**	**95.45**
10 March, *m* = 6	1.98	30.83	**95.45**
10 March, *m* = 8	**1.85**	29.96	**95.45**
19 March, *m* = 3	**2.18**	90.09	86.96
19 March, *m* = 6	2.77	91.29	**91.30**
19 March, *m* = 8	2.36	**69.68**	**91.30**

## Data Availability

The data and the code for this tutorial paper are publicly available on the Github repository https://github.com/atariq2891/Reporting-delay-adjustment-code/tree/main (accessed on 8 August 2025).

## Supplementary Materials

The following supporting information can be downloaded at: https://www.mdpi.com/article/10.3390/v17121598/s1, Figure S1: Reporting delay distribution for each WHO Situation Report (33–61) based on reporting delay adjustment by date of symptom onset. Figure S2: Reporting delay-adjusted local incidence for the COVID-19 outbreak in Singapore as of 17 March 2020; Figure S3: Reporting delay-adjusted incidence for the COVID-19 outbreak in Chile as of 27 March 2020.
