# Supplementary material for: Nowcast-It: A Practical Toolbox for Real-Time Adjustment of Reporting Delays in Epidemic Surveillance
Source: Viruses. 2025 Dec 10;17(12):1598. doi: 10.3390/v17121598 (PMC12737790; doi:10.3390/v17121598)
Supplement: Supplementary file 1 [file viruses-17-01598-s001.zip › viruses-3856175-supplementary.pdf]

Supplementary file

Nowcast-It: A Practical Toolbox for Real-Time Adjustment of Reporting Delays in Epidemic Surveillance

Amna Tariq [1,*], Ping Yan [2], Amanda Bleichrodt [3] and Gerardo Chowell [4,5,*]

[1] Department of Pediatrics Infectious Diseases, Stanford School of Medicine, Palo Alto, CA
[2] Department of Statistics and Actuarial Science, University of Waterloo, Ottawa, Canada
[3] Department of Public Health Sciences, Clemson University, Clemson, SC
[4] Department of Population Health Sciences, Georgia State University, Atlanta, GA
[5] Department of Applied Mathematics, Kyung Hee University, Yongin 17104, Korea
* Correspondence: atariq1@stanford.edu (AT); gchowell@gsu.edu (GC)

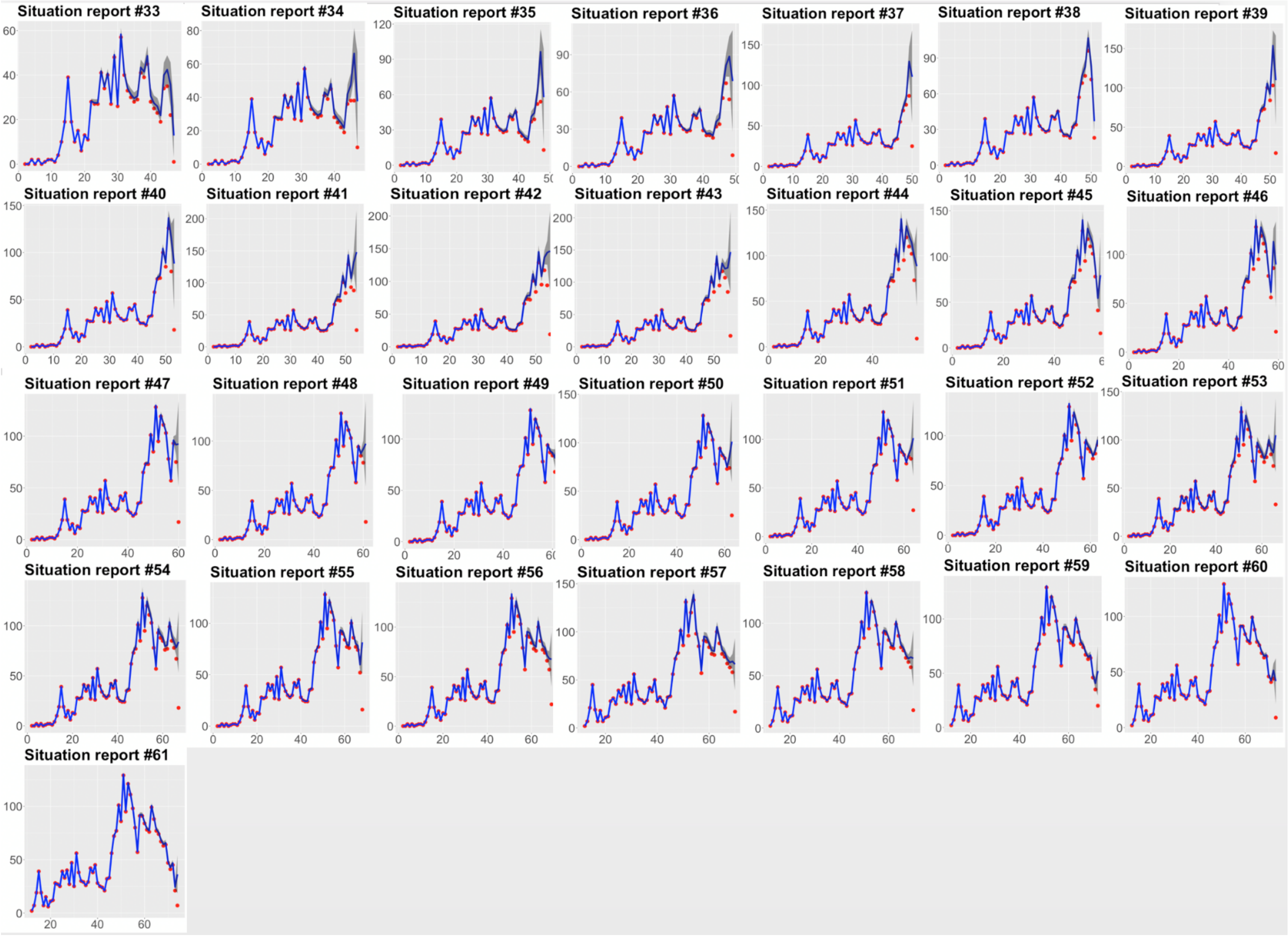

Figure S1: Reporting delay distribution for each WHO Situation Report (33 – 61) based on reporting delay adjustment by date of symptom onset. Reported incidence data (red points) are plotted with the mean estimate of the reporting delay adjusted incidence (solid blue line) and the associated 95% prediction intervals (dark grey shaded region)[1].

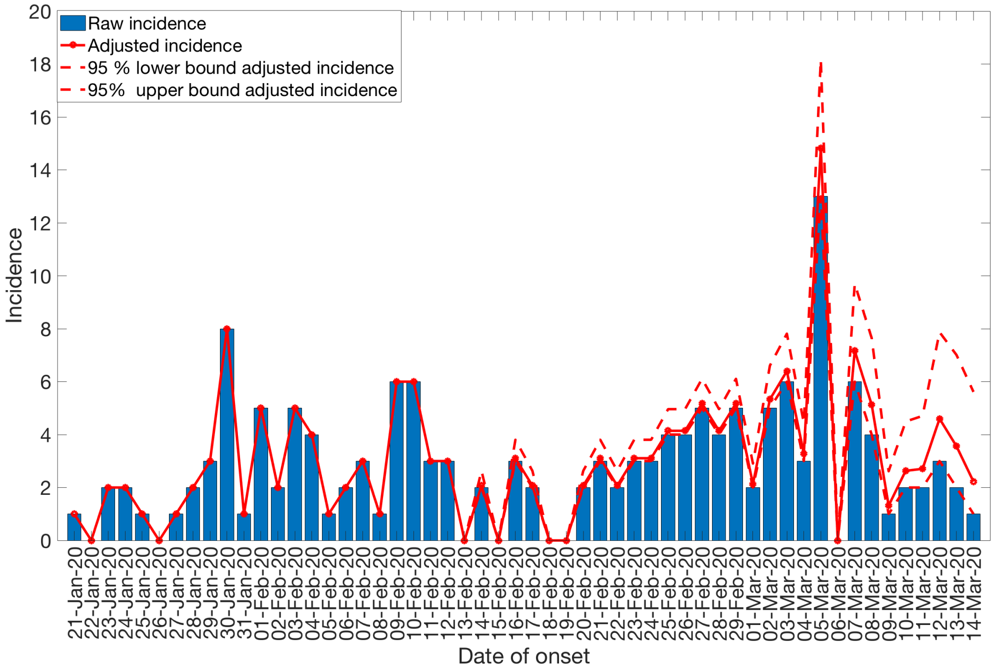

Figure S2: Reporting delay adjusted local incidence for the COVID-19 outbreak in Singapore as of March 17, 2020. Blue bars represent the raw incidence, red solid line represents the adjusted incidence, and red dotted lines represent the 95% lower and upper bound of the adjusted incidence[2].

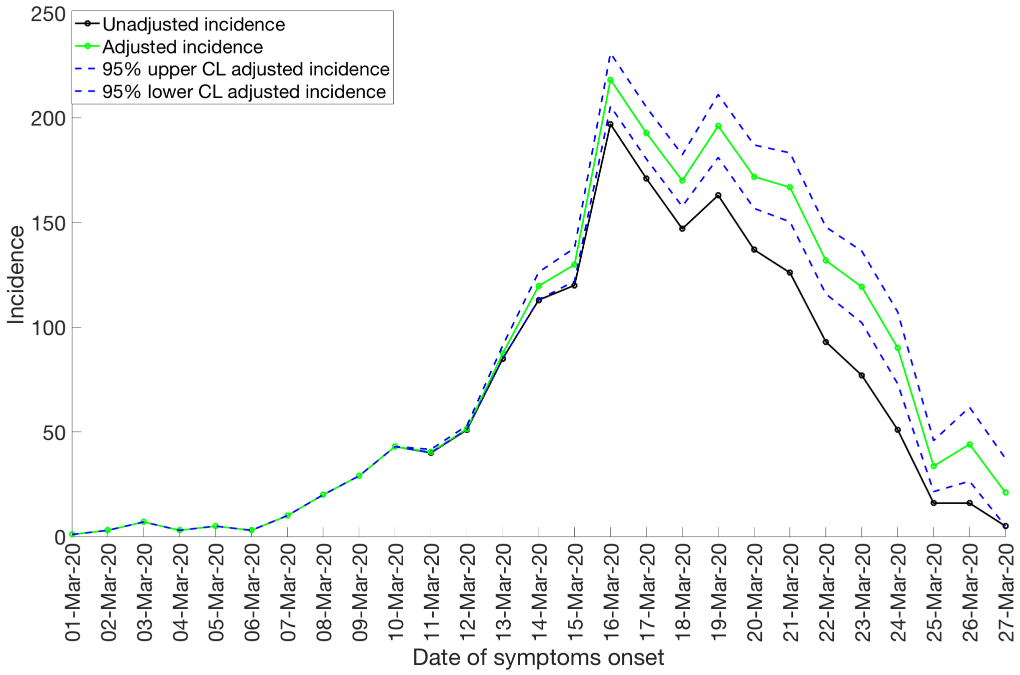

Figure S3: Reporting delay adjusted incidence for the COVID-19 outbreak in Chile as of March 27, 2020. Black line with circles represent the raw incidence, green solid line with circles

represents the adjusted incidence, and blue dotted lines represent the 95% lower and upper bound of the adjusted incidence

References

1. Roosa K, Tariq A, Yan P, Hyman JM, Chowell G. Multi-model forecasts of the ongoing Ebola epidemic in the Democratic Republic of Congo, March–October 2019. Journal of The Royal Society Interface. 2020;17(169):20200447.doi:10.1098/rsif.2020.0447
2. Tariq A, Lee Y, Roosa K, Blumberg S, Yan P, Ma S, et al. Real-time monitoring the transmission potential of COVID-19 in Singapore, March 2020. BMC Medicine. 2020;18(1):166.10.1186/s12916-020-01615-9
